# Comparative analysis of differential network modularity in tissue specific normal and cancer protein interaction networks

**DOI:** 10.1186/2043-9113-3-19

**Published:** 2013-10-06

**Authors:** Md Fahmid Islam, Md Moinul Hoque, Rajat Suvra Banik, Sanjoy Roy, Sharmin Sultana Sumi, F M Nazmul Hassan, Md Tauhid Siddiki Tomal, Ahmad Ullah, K M Taufiqur Rahman

**Affiliations:** 1 Biotechnology and Genetic Engineering Discipline, Khulna University, Khulna 9208, Bangladesh; 2 Forestry and Wood Technology Discipline, Khulna University, Khulna 9208, Bangladesh

**Keywords:** Cancer, Signal transduction pathway, Network biology, Protein interaction network, Molecular complex, Crucial node, Overlapping module

## Abstract

**Background:**

Large scale understanding of complex and dynamic alterations in cellular and subcellular levels during cancer in contrast to normal condition has facilitated the emergence of sophisticated systemic approaches like network biology in recent times. As most biological networks show modular properties, the analysis of differential modularity between normal and cancer protein interaction networks can be a good way to understand cancer more significantly. Two aspects of biological network modularity e.g. detection of molecular complexes (potential modules or clusters) and identification of crucial nodes forming the overlapping modules have been considered in this regard.

**Methods:**

In the current study, the computational analysis of previously published protein interaction networks (PINs) has been conducted to identify the molecular complexes and crucial nodes of the networks. Protein molecules involved in ten major cancer signal transduction pathways were used to construct the networks based on expression data of five tissues e.g. bone, breast, colon, kidney and liver in both normal and cancer conditions. MCODE (molecular complex detection) and ModuLand methods have been used to identify the molecular complexes and crucial nodes of the networks respectively.

**Results:**

In case of all tissues, cancer PINs show higher level of clustering (formation of molecular complexes) than the normal ones. In contrast, lower level modular overlapping is found in cancer PINs than the normal ones. Thus a proposition can be made regarding the formation of some giant nodes in the cancer networks with very high degree and resulting in reduced overlapping among the network modules though the predicted molecular complex numbers are higher in cancer conditions.

**Conclusion:**

The study predicts some major molecular complexes that might act as the important regulators in cancer progression. The crucial nodes identified in this study can be potential drug targets to combat cancer.

## Background

Reductionist philosophy has directed biological research for decades [1,2]. A significant amount of information has been generated so far in the field of biological sciences as enrichment of human knowledgebase to understand life [1]. Despite enormous success of reductionism to decode the structural and functional attributes at cellular and molecular levels of life-organization, it is progressively becoming clearer that biological functions can rarely be credited to discrete perception of individual molecules. Alternatively, most biological phenomena emerge due to extremely interactive complexity derived from functional integrity of cell’s numerous constituents [2]. Various recent approaches have been initiated and accomplished to study biological systems in more integrative and comprehensive way. Network model can play an important role to understand the complex network system based on multiple sets of interactions and to make plain and clear analysis of the origin of observed network characteristics [3-7]. Network biology has thus come out at present time as a revolutionary approach for the empirical study to understand complex biological systems [3,8-12].

In cancer condition, genomic instability results in alterations of downstream signal transduction pathways and protein-protein interactions. Current understanding of the dynamic changes at genomic and proteomic levels indicates that cancer can be considered as a stochastic phenomenon rather than being the result of some specific linear alterations [13]. Insightful understanding of comparative regulatory patterns in normal and cancerous cells requires in detailed study of molecular interactions [14] and network biology has prospective usefulness in this regard [15]. The concepts of network biology can be utilized to decipher the differential interaction patterns between normal and cancer conditions through construction of biomolecular networks and subsequent in depth analysis of the networks.

Studying modularity of biomolecular networks can be an efficient way to understand their inherent properties and identify the crucial molecular sets and components of the networks (which is a basic challenge of the study of these networks) [16]. In most of the cases biomolecular networks show modular organization that means the network can be divided into modules according to the density of connections among the nodes of a network. More specifically, modules are the subsets of a network that have comparatively high connectedness among the nodes (through the edges) forming the modules. The modules have lots of connections within themselves but sparse connections among them [17,18]. From a general point of view, depiction of the modules is useful in understanding the structural and functional features of networks, which has stimulated many empirical researches as well as practical applications e.g. protein complex and drug target identification [19,20].

The main objective of this paper was to study the differential modularity patterns of normal and cancer protein interaction networks (PINs). The PINs were constructed for five tissues e.g. bone, breast, colon, kidney and liver in both normal and cancer conditions [21]. The network construction was based on expression data of protein molecules participating in ten major cancer signal transduction pathways. MCODE (molecular complex detection) [22] method was used to identify and analyze potential molecular complexes (modules or clusters) of the networks. Another method ModuLand [23,24] was used for identification and subsequent analysis of crucial nodes forming overlapping modules of the networks.

## Methods

The primary data required were retrieved from differential expression database GeneHub-GEPIS (an online bioinformatics tool for inferring gene expression patterns in a large panel of normal and cancer tissues; http://research-public.gene.com/Research/genentech/genehub-gepis/index.html) [25] and protein-protein interaction prediction tools e.g. PIPs (Human Protein-Protein Interaction Prediction; http://www.compbio.dundee.ac.uk/www-pips/) [26,27] and STRING (a database of known and predicted protein interactions; http://string.embl.de/) [28-33]. Cytoscape software package [34-36] was used to construct protein interaction networks (PINs) (Additional files 1 and 2) [21]. For modularity analysis two Cytoscape plugins namely MCODE and ModuLand were used. MCODE was used to identify and rank all possible molecular complexes of particular networks and ModuLand was used to identify crucial nodes forming the overlapping modules in those networks. MCODE detects densely connected regions in large protein interaction networks, which may be characterized as molecular complexes [22]. The MCODE method stands on vertex weighting by local neighborhood density and outward traversal from a locally dense seed protein to isolate the dense regions according to given parameters. The ModuLand method provides an algorithm for determining extensively overlapping network modules [23,24]. Additionally, it identifies several hierarchical layers of modules through representation of modules of the lower layer by meta-nodes of the higher hierarchical layer. This method predicts the function of the whole module and determines key nodes bridging two or multiple modules through assigning module cores.

During MCODE and ModuLand analysis default parameter values were utilized. The default MCODE set up was fixed like, Find Clusters: in Whole Network; Network Scoring (Advanced Option)- a) Include Loops: Turn off, b) Degree Cutoff: 2; Cluster Finding- a) Haircut: Turn on, b) Fluff: Turn off, c) Node Score Cutoff: 0.2, d) K-Core: 2, e) Max. Depth: 100. During ModuLand analysis, selected unweighted network option was taken with default value 1. ModuLand was run to identify and visualize overlapping modules and merged (for modules) with threshold value 1.0 to create correlation matrix of original modularization and module correlation histogram. Measures option of ModuLand was used to calculate the graph related parameters of the overlapping modules.

## Results and discussion

### Molecular complex detection

**Figure 1:**
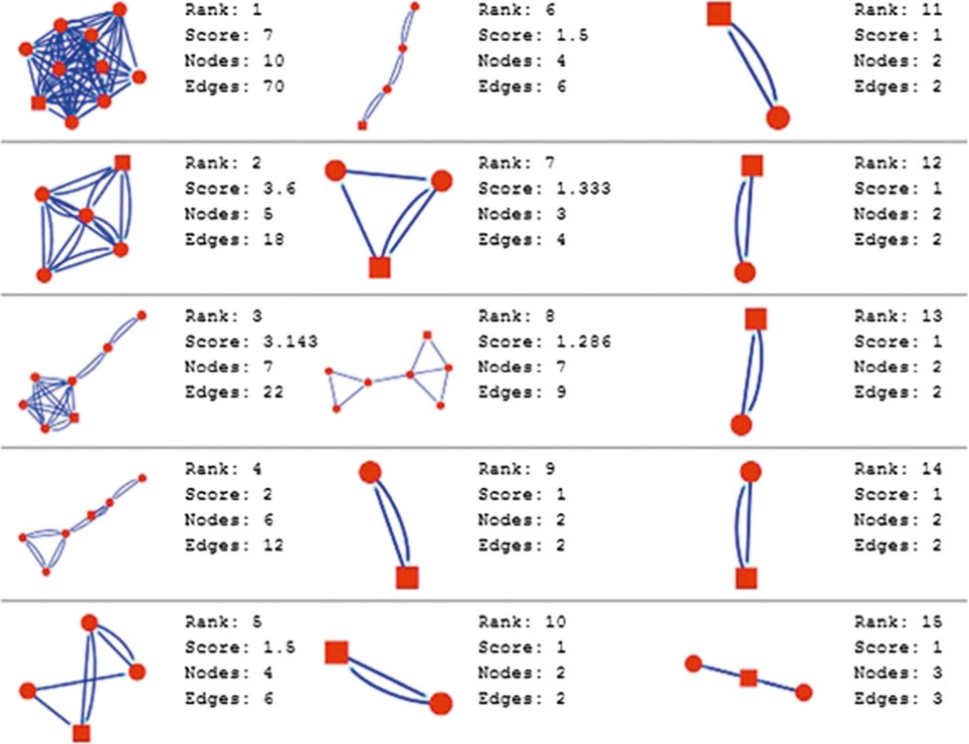
MCODE analysis of normal condition protein interaction network in bone.

**Figure 2:**
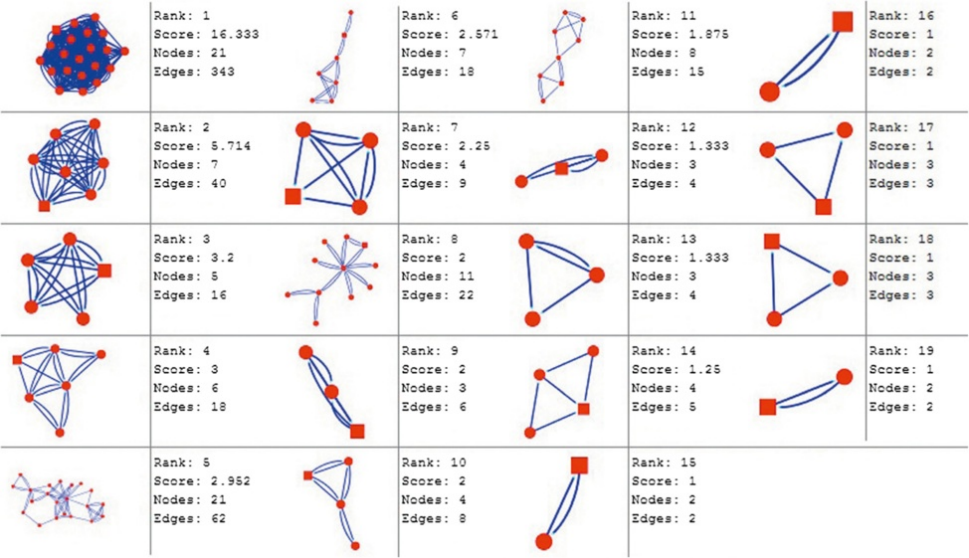
MCODE analysis of cancer condition protein interaction network in bone.

**Figure 3:**
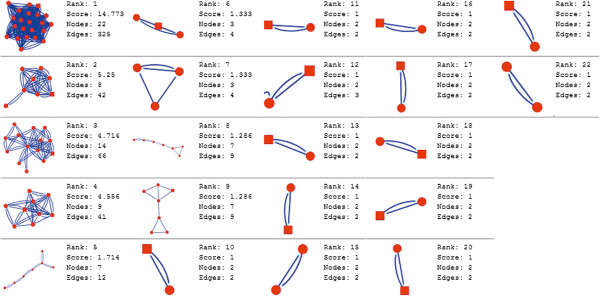
MCODE analysis of normal condition protein interaction network in breast.

**Figure 4:**
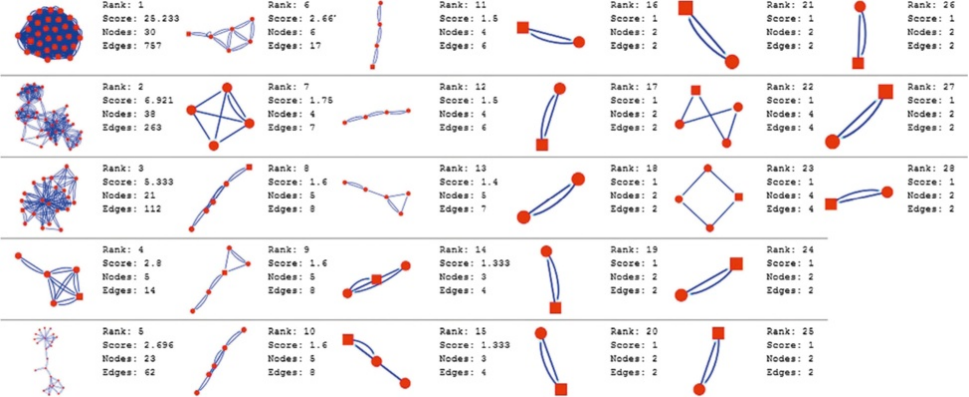
MCODE analysis of cancer condition protein interaction network in breast.

**Figure 5:**
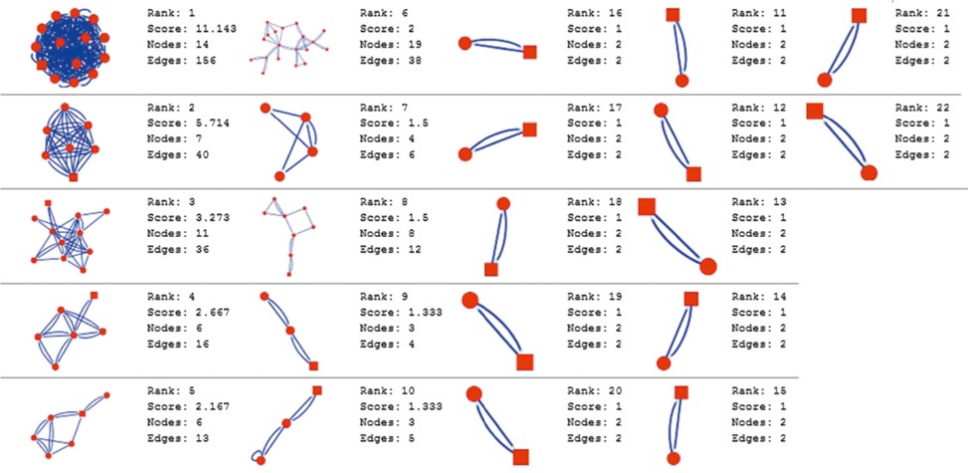
MCODE analysis of normal condition protein interaction network in colon.

**Figure 6:**
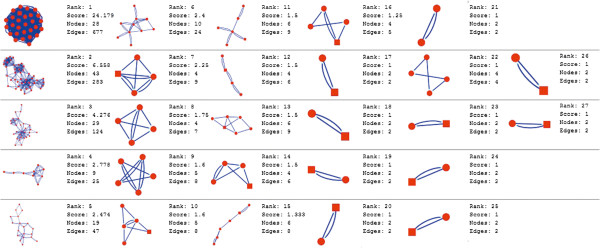
MCODE analysis of cancer condition protein interaction network in colon.

**Figure 7:**
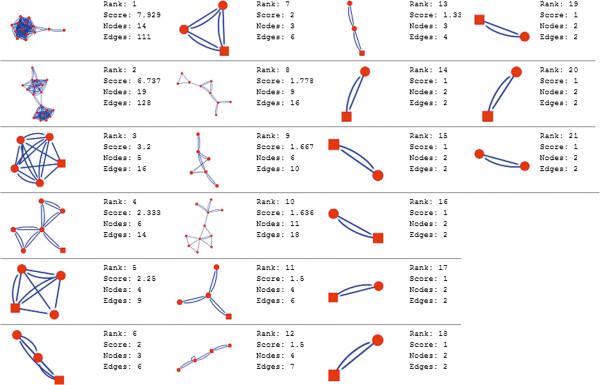
MCODE analysis of normal condition protein interaction network in kidney.

**Figure 8:**
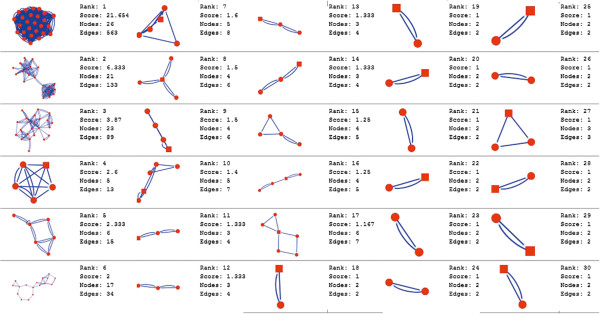
MCODE analysis of cancer condition protein interaction network in kidney.

**Figure 9:**
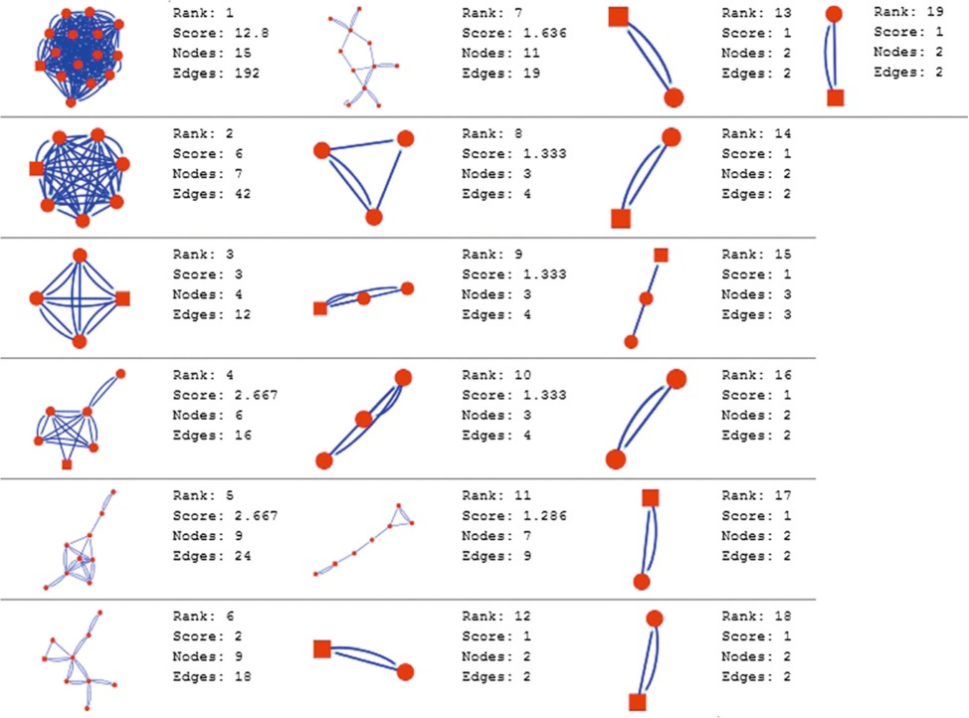
MCODE analysis of normal condition protein interaction network in liver.

**Figure 10:**
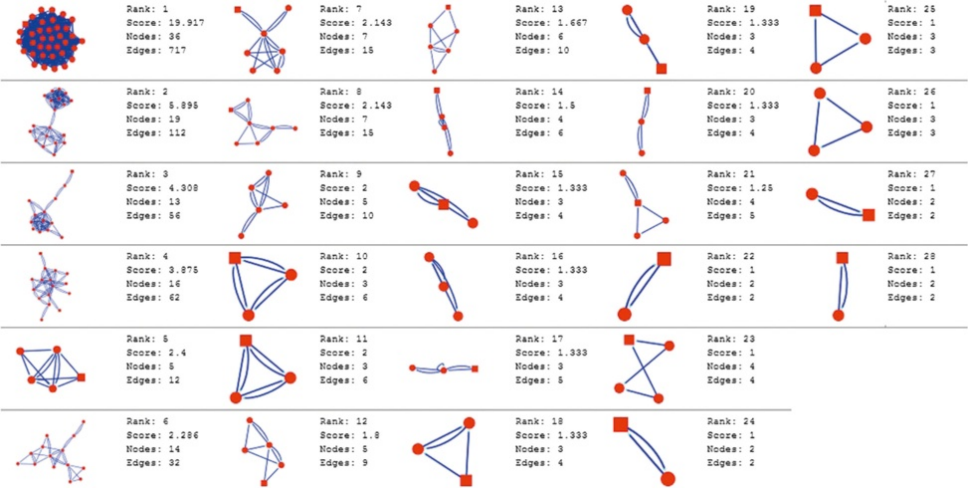
MCODE analysis of cancer condition protein interaction network in liver.

Molecular complex detection (MCODE) method has been used to evaluate yeast protein interaction compilation using known molecular complex data from mass spectrometry of the proteome [19,37]. This leads to the observation that highly interconnected, or dense regions of the network may represent molecular complexes [38]. The numbers of possible modules that can be said as molecular complexes, differ between normal and cancer conditions in each of the five tissues (Figures 1, 2, 3, 4, 5, 6, 7, 8, 9 and 10). The ranked molecular complex numbers of normal and cancer protein interaction networks are 15 and 19 for bone, 22 and 28 for breast, 22 and 27 for colon, 21 and 30 for kidney and 19 and 28 for liver respectively (Figures 1, 2, 3, 4, 5, 6, 7, 8, 9 and 10). In all cases, possible molecular complex numbers increase in cancer condition. The statistical significance test also supports the difference (at *p* ≤ 0.05) and depicts that the molecular complex numbers of cancer PINs are significantly increased than the normal PINs (at *p* = 0.02) (Additional file 3).

Kidney cancer shows highest increment during cancer in comparison to normal state for predicted molecular complex numbers (Figures 7 and 8). Not only the molecular complex numbers, all other parameters e.g. scores, nodes and edges of the molecular complex networks differ between normal and cancer conditions for each tissue (Figures 1, 2, 3, 4, 5, 6, 7, 8, 9 and 10 and Additional file 4).

As in case of cancer networks, the related edge and node numbers increase from the normal conditions for all five tissues, the overall clustering is also enhanced in cancer networks. The normal and cancer networks were mainly constructed based on the expression and interaction data of protein molecules participating in major cancer signal transduction pathways which has been described in our previous paper [21]. The event of increased edges and nodes in cancer tissues compared with normal tissues can be explained as the enhancement of molecular interactions at proteomic level in cancer states in comparison to normal states. It is mentionable that the graphical representations of such differences are based on already validated experimental data regarding gene expression and protein interaction. The biological meaning of the observed differences seems to be very obvious indicating that cancer tissue involves more proteins to interact with each other during cancer signaling.

A current report supports that disease genes tend to have higher degree and connectivity in comparison to non-disease genes in terms of expression and interaction of proteins [39]. Some studies also indicate that proteins encoded by cancer genes can interact strongly with other proteins and show higher connectivity than normal condition [40]. There is also evidence of overrepresentation of 10% of protein interaction clusters within the cancer interactome when compared to the normal protein interaction networks [7].

### Overlapping module and crucial node identification from the networks

**Figure 11:**
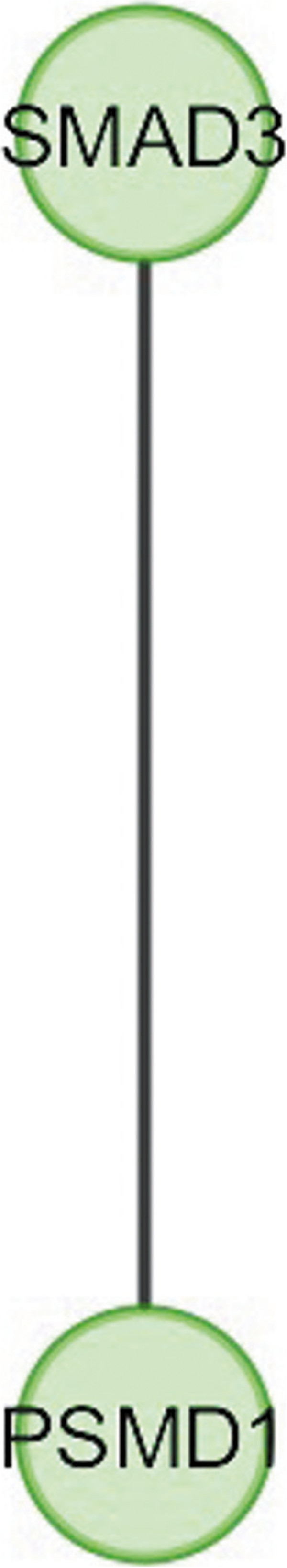
Overlapping module in normal condition of bone.

**Figure 12:**
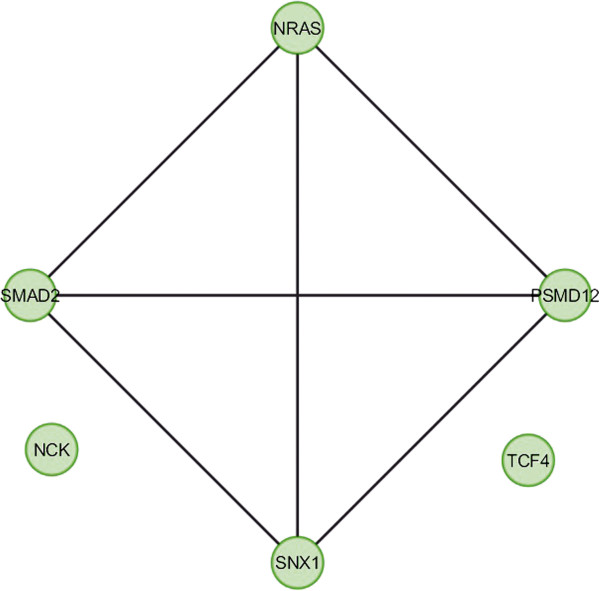
Overlapping module in normal condition of breast.

**Figure 13:**
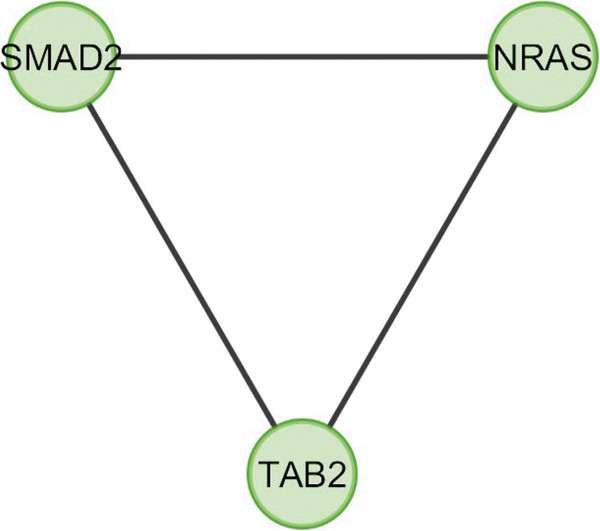
Overlapping module in cancer condition of breast.

**Figure 14:**
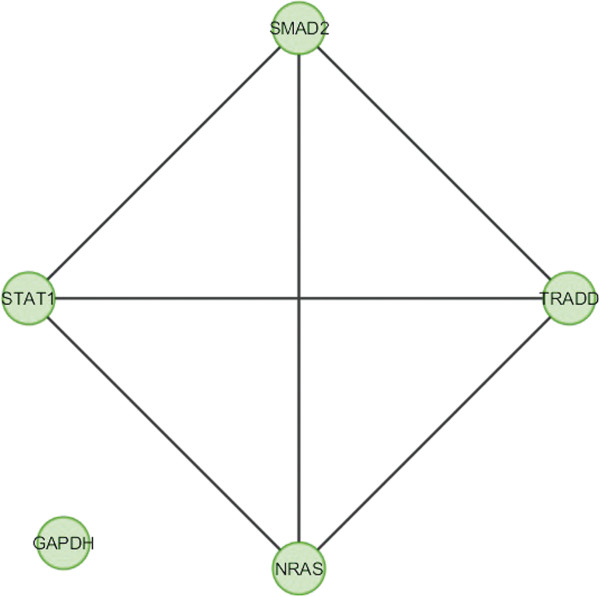
Overlapping module in normal condition of colon.

**Figure 15:**
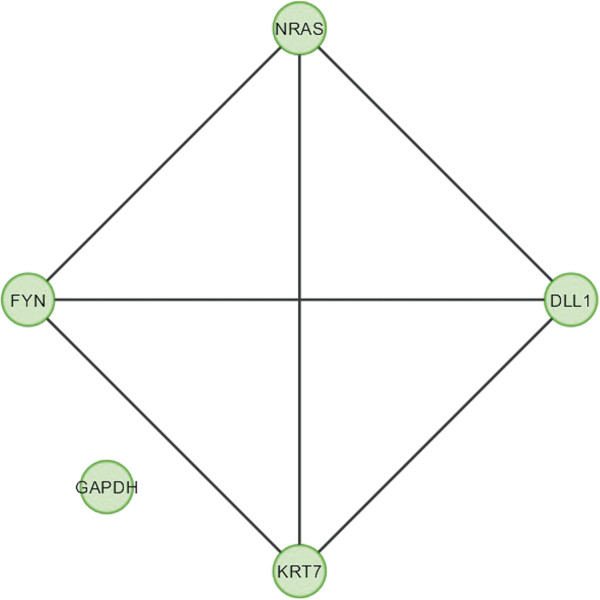
Overlapping module in cancer condition of colon.

**Figure 16:**
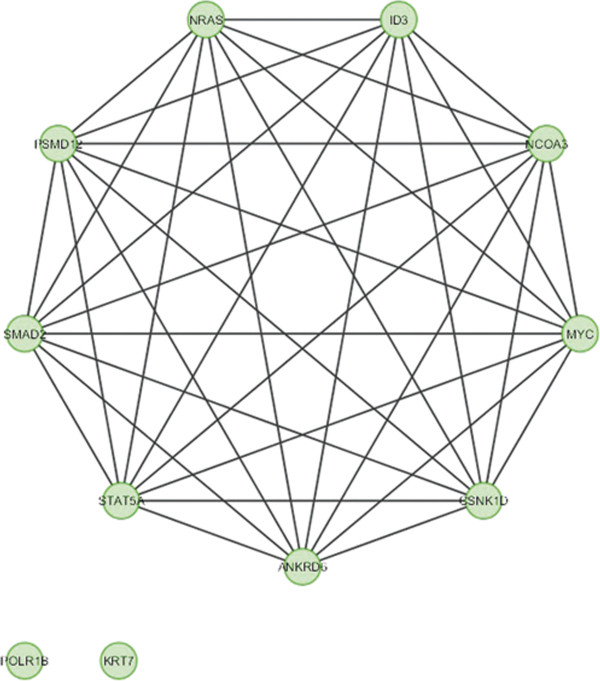
Overlapping module in normal condition of kidney.

**Figure 17:**
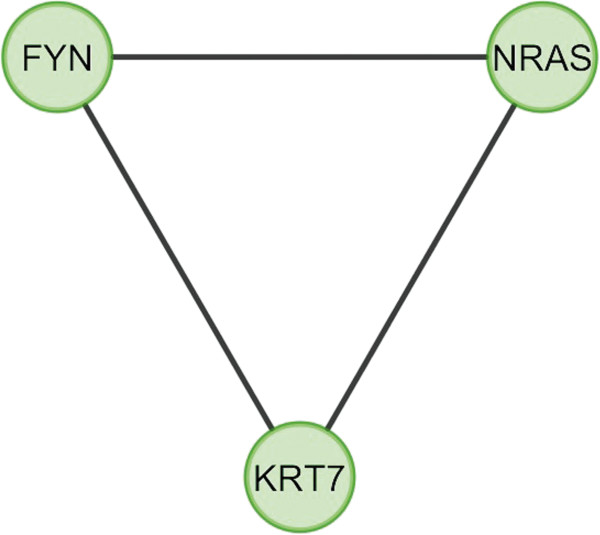
Overlapping module in cancer condition of kidney.

**Figure 18:**
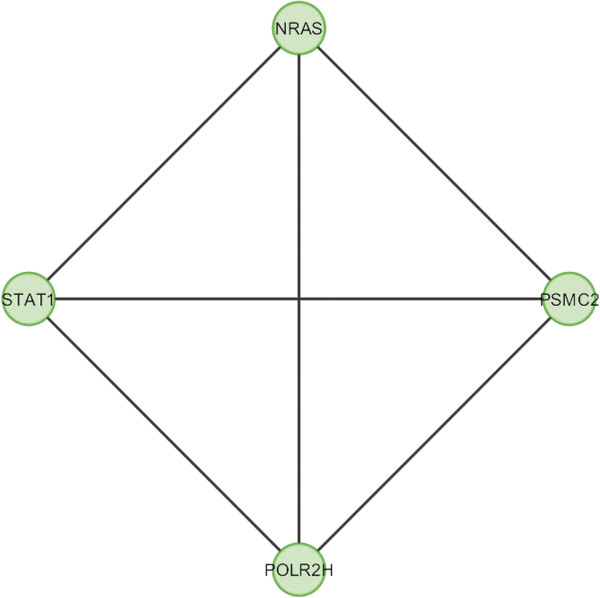
Overlapping module in normal condition of liver.

**Figure 19:**
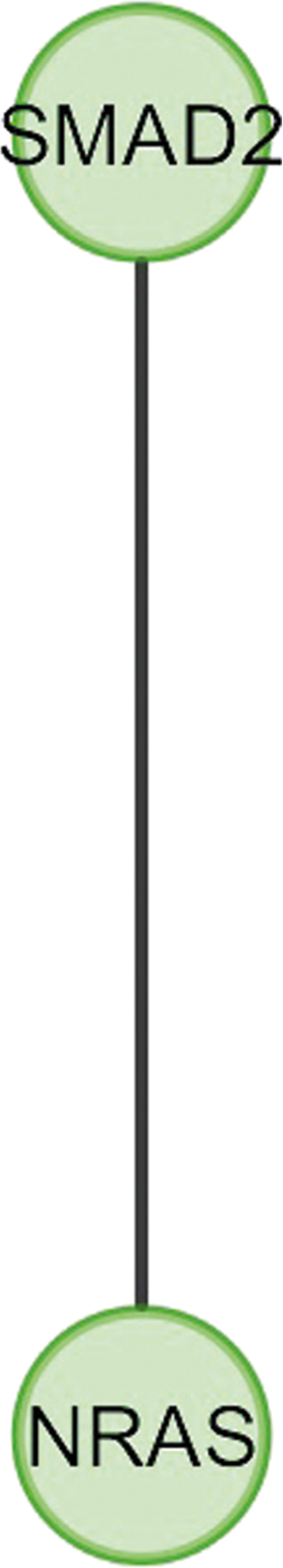
Overlapping module in cancer condition of liver.

In case of bone, overlapping module is present in normal condition but absent in cancer (Figure 11). Overlapping modules between normal and cancer states differ for all other tissues (Figures 12, 13, 14, 15, 16, 17, 18 and 19). In breast, kidney and liver edge and node numbers decrease in cancer and most of the molecules forming the overlapping networks are changed (Figures 12, 13, 16, 17, 18 and 19). In colon, edge and node numbers remain constant but most of the molecules forming the overlapping modules are altered (Figures 14, 15). The highest fluctuation of overlapping module from the point of node and edge number and molecules forming the overlapping networks occurs in case of kidney (Figures 16, 17). The nodes of the overlapping module can be said as the crucial nodes with module centrality (which is the central node of the related modules formed by ModuLand) of the respective network [41]. The important network properties of the overlapping modules have been compared in Tables 1, 2, 3, 4, 5, 6, 7, 8 and 9.

**Table 1:** Graph related parameters for normal condition of bone

**NodeID**	**Effective degree**	**ModuLand community centrality**	**Betweenness centrality**	**ModuLand overlap**	**ModuLand bridgeness**
SMAD3	1	32.76882	0	1	0
PSMD1	1	32.76882	0	1	0

**Table 2:** Graph related parameters for normal condition of breast

**NodeID**	**Effective degree**	**ModuLand community centrality**	**Betweenness centrality**	**ModuLand overlap**	**ModuLand bridgeness**
SMAD2	2.579363	564.9435	0	1	0
NRAS	1.450132	87.90458	0	1	0
PSMD12	2.022537	464.4288	0	1	0
SNX1	2.999741	13.47424	0	1	0
TCF4	0	0	0	1	0
NCK	0	0	0	1	0

**Table 3:** Graph related parameters for cancer condition of breast

**NodeID**	**Effective degree**	**ModuLand community centrality**	**Betweenness centrality**	**ModuLand overlap**	**ModuLand bridgeness**
SMAD2	2	325.3334	0	1	0
NRAS	2	79.67153	0	1	0
TAB2	2	245.9151	0	1	0

**Table 4:** Graph related parameters for normal condition of colon

**NodeID**	**Effective degree**	**ModuLand community centrality**	**Betweenness centrality**	**ModuLand overlap**	**ModuLand bridgeness**
STAT1	2.239262	6792.052	0	1	0
SMAD2	2.650014	6520.055	0	1	0
TRADD	2.68063	255.6004	0	1	0
NRAS	1.443442	91.6213	0	1	0
GAPDH	0	0	0	1	0

**Table 5:** Graph related parameters for cancer condition of colon

**NodeID**	**Effective degree**	**ModuLand community centrality**	**Betweenness centrality**	**ModuLand overlap**	**ModuLand bridgeness**
FYN	2.792082	486.4563	0	1	0
NRAS	2.049608	130.5231	0	1	0
DLL1	1.730402	326.6208	0	1	0
KRT7	1.003324	29.61218	0	1	0
GAPDH	0	0	0	1	0

**Table 6:** Graph related parameters for normal condition of kidney

**NodeID**	**Effective degree**	**ModuLand community centrality**	**Betweenness centrality**	**ModuLand overlap**	**ModuLand bridgeness**
STAT5A	2.657912	51156.29	0	1	0
SMAD2	6.401464	50932.09	0	1	0
PSMD12	5.006495	1121.599	0	1	0
NRAS	6.620978	168.6027	0	1	0
ID3	4.308711	264.494	0	1	0
NCOA3	7.118026	258.9439	0	1	0
MYC	3.992952	421.0773	0	1	0
CSNK1D	2.100971	121.4912	0	1	0
ANKRD6	2.618028	66.80382	0	1	0
KRT7	0	0	0	1	0
POLR1B	0	0	0	1	0

**Table 7:** Graph related parameters for cancer condition of kidney

**NodeID**	**Effective degree**	**ModuLand community centrality**	**Betweenness centrality**	**ModuLand overlap**	**ModuLand bridgeness**
FYN	2	125.7711	0	1	0
NRAS	2	107.3406	0	1	0
KRT7	2	18.65667	0	1	0

**Table 8:** Graph related parameters for normal condition of liver

**NodeID**	**Effective degree**	**ModuLand community centrality**	**Betweenness centrality**	**ModuLand overlap**	**ModuLand bridgeness**
STAT1	2.86861	652.5232	0	1	0
NRAS	2.303882	214.573	0	1	0
PSMC2	2.009952	394.8492	0	1	0
POLR2H	2.006563	67.90855	0	1	0

**Table 9:** Graph related parameters for cancer condition of liver

**NodeID**	**Effective degree**	**ModuLand community centrality**	**Betweenness centrality**	**ModuLand overlap**	**ModuLand bridgeness**
SMAD2	1	105.1724	0	0	1
NRAS	1	105.1724	0	0	1

**Figure 20:**
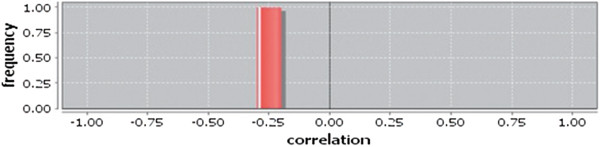
Correlation histogram for normal condition of bone.

**Figure 21:**
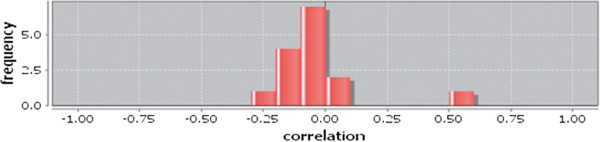
Correlation histogram for normal condition of breast.

**Figure 22:**
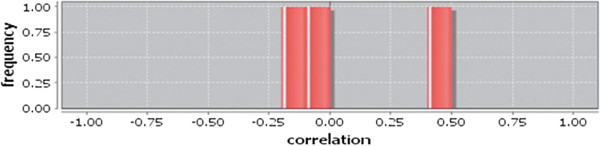
Correlation histogram for cancer condition of breast.

**Figure 23:**
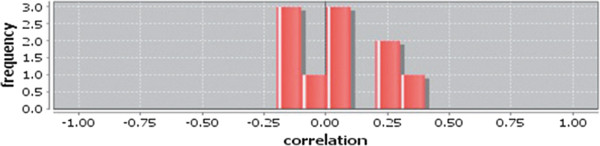
Correlation histogram for normal condition of colon.

**Figure 24:**
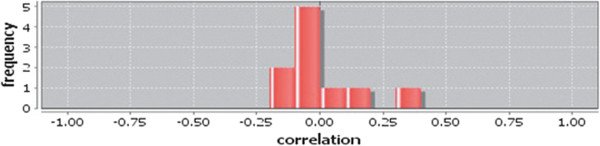
Correlation histogram for cancer condition of colon.

**Figure 25:**
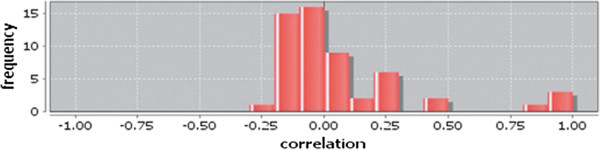
Correlation histogram for normal condition of kidney.

**Figure 26:**
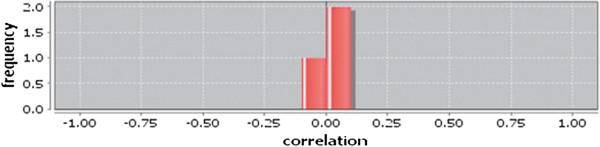
Correlation histogram for cancer condition of kidney.

**Figure 27:**
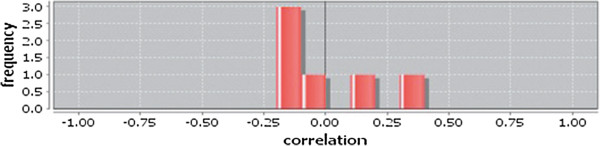
Correlation histogram for normal condition of liver.

**Figure 28:**
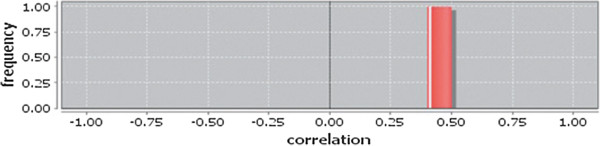
Correlation histogram for cancer condition of liver.

Correlation matrix and correlation histogram in both normal and cancer conditions for each tissue represent the nature of correlation among the nodes of the overlapping modules (Tables 10, 11, 12, 13, 14, 15, 16, 17 and 18 and Figures 20, 21, 22, 23, 24, 25, 26, 27 and 28). Correlation matrix represents all the possible interactions of the overlapping modules. Correlation histogram represents only the valid interactions at certain threshold (here 1.0). From the correlation matrix and histogram, it is found that the interactions among the nodes of overlapping modules differ between normal and cancer cases (Tables 10, 11, 12, 13, 14, 15, 16, 17 and 18 and Figures 20, 21, 22, 23, 24, 25, 26, 27 and 28). The statistical significance test also supports the difference (at *p* ≤ 0.1) and depicts that valid interactions (at threshold 1.0) of overlapping modules in cancer PINs are significantly increased than the normal PINs (at *p* = 0.08) (Additional file 3).

**Table 10:** Correlation matrix for normal condition of bone

	**SMAD3**	**PSMD1**
SMAD3	1	-0.24068
PSMD1	-0.24068	1

**Table 11:** Correlation matrix for normal condition of breast

	**SMAD2**	**NRAS**	**PSMD12**	**SNX1**	**TCF4**	**NCK**
SMAD2	1	-0.03436	-0.20478	0.024483	-0.13341	-0.13341
NRAS	-0.03436	1	0.520993	0.053684	-0.11923	-0.11923
PSMD12	-0.20478	0.520993	1	-0.0323	-0.09971	-0.09971
SNX1	0.024483	0.053684	-0.0323	1	-0.01227	-0.01227
TCF4	-0.13341	-0.11923	-0.09971	-0.01227	1	-0.00608
NCK	-0.13341	-0.11923	-0.09971	-0.01227	-0.00608	1

**Table 12:** Correlation matrix for cancer condition of breast

	**SMAD2**	**NRAS**	**TAB2**
SMAD2	1	-0.0843	-0.14403
NRAS	-0.0843	1	0.453856
TAB2	-0.14403	0.453856	1

**Table 13:** Correlation matrix for normal condition of colon

	**STAT1**	**SMAD2**	**TRADD**	**NRAS**	**GAPDH**
STAT1	1	0.345955	0.03459	0.01538	-0.1398
SMAD2	0.345955	1	0.219089	0.011785	-0.13841
TRADD	0.03459	0.219089	1	0.252983	-0.11925
NRAS	0.01538	0.011785	0.252983	1	-0.09299
GAPDH	-0.1398	-0.13841	-0.11925	-0.09299	1

**Table 14:** Correlation matrix for cancer condition of colon

	**FYN**	**NRAS**	**DLL1**	**KRT7**	**GAPDH**
FYN	1	-0.03623	-0.11094	-0.09958	-0.10397
NRAS	-0.03623	1	0.302796	0.126064	-0.09173
DLL1	-0.11094	0.302796	1	0.014064	-0.06608
KRT7	-0.09958	0.126064	0.014064	1	-0.01448
GAPDH	-0.10397	-0.09173	-0.06608	-0.01448	1

**Table 15:** Correlation matrix for normal condition of kidney

	**STAT5A**	**SMAD2**	**PSMD12**	**NRAS**	**ID3**	**NCOA3**	**MYC**	**CSNK1D**	**ANKRD6**	**KRT7**	**POLR1B**
STAT5A	1	0.865947	0.066442	0.144672	0.035023	0.026829	0.039755	-0.17473	-0.22182	-0.13318	-0.16338
SMAD2	0.865947	1	0.085819	0.110893	0.020009	0.026779	0.026134	-0.1123	-0.1689	-0.13318	-0.16338
PSMD12	0.066442	0.085819	1	0.491691	0.276862	0.255873	0.253694	-0.04079	-0.16593	-0.12014	-0.14737
NRAS	0.144672	0.110893	0.491691	1	0.275515	0.246432	0.265387	-0.06826	-0.14946	-0.09565	-0.11733
ID3	0.035023	0.020009	0.276862	0.275515	1	0.983226	0.98228	-0.02846	-0.02835	-0.08511	-0.1044
NCOA3	0.026829	0.026779	0.255873	0.246432	0.983226	1	0.987846	0.013256	-0.0074	-0.08704	-0.10677
MYC	0.039755	0.026134	0.253694	0.265387	0.98228	0.987846	1	-0.01465	-0.02672	-0.0882	-0.1082
CSNK1D	-0.17473	-0.1123	-0.04079	-0.06826	-0.02846	0.013256	-0.01465	1	0.422071	-0.02188	-0.02685
ANKRD6	-0.22182	-0.1689	-0.16593	-0.14946	-0.02835	-0.0074	-0.02672	0.422071	1	-0.02344	-0.02876
KRT7	-0.13318	-0.13318	-0.12014	-0.09565	-0.08511	-0.08704	-0.0882	-0.02188	-0.02344	1	-0.00784
POLR1B	-0.16338	-0.16338	-0.14737	-0.11733	-0.1044	-0.10677	-0.1082	-0.02685	-0.02876	-0.00784	1

**Table 16:** Correlation matrix for cancer condition of kidney

	**FYN**	**NRAS**	**KRT7**
FYN	1	0.094025	-0.08621
NRAS	0.094025	1	0.062247
KRT7	-0.08621	0.062247	1

**Table 17:** Correlation matrix for normal condition of liver

	**STAT1**	**NRAS**	**PSMC2**	**POLR2H**
STAT1	1	-0.00568	-0.14947	-0.19596
NRAS	-0.00568	1	0.374997	-0.17413
PSMC2	-0.14947	0.374997	1	0.173069
POLR2H	-0.19596	-0.17413	0.173069	1

**Table 18:** Correlation matrix for cancer condition of liver

	**SMAD2**	**NRAS**
SMAD2	1	0.438329
NRAS	0.438329	1

In case of bone, there is no correlation matrix and correlation histogram for cancer as there is no overlapping module (Table 10; Figure 20). Correlation matrix and correlation histogram show reduced number of interactions during cancer in case of breast, kidney and liver (Tables 11, 12, 15, 16, 17, and 18; Figures 21, 22, 25, 26, 27 and 28). In case of colon, the interaction number remains the same (Tables 13, 14; Figures 23, 24). The correlation frequency in the histograms fluctuates between two conditions as the molecules representing the nodes of overlapping modules differ (Figures 20, 21, 22, 23, 24, 25, 26, 27 and 28).

The crucial nodes identified from the overlapping modules are found to show important biological signification in recently reconstructed high-quality *Staphylococcus aureus* metabolic network model [41-43]. Identification of functional subgraphs from cancer protein interaction networks representing the important modules and their components has been a key issue in some papers [44,45].

The parameter values used for MCODE and ModuLand analysis remained the same for both normal and cancer state study and were applied according to the suggested range by plugin developers. So it can be assumed that the parameter values have not any significant effect on the conclusions. It can be also said that some minor effects of parameter values may have some influence but these will not affect our understanding of qualitative comparison between normal and cancer PINs.

**Figure 29:**
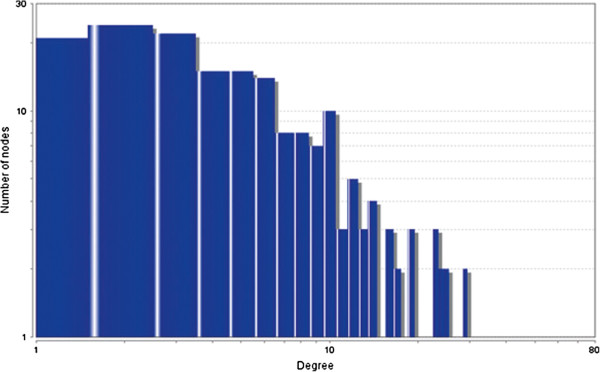
Degree distribution in bone normal network.

**Figure 30:**
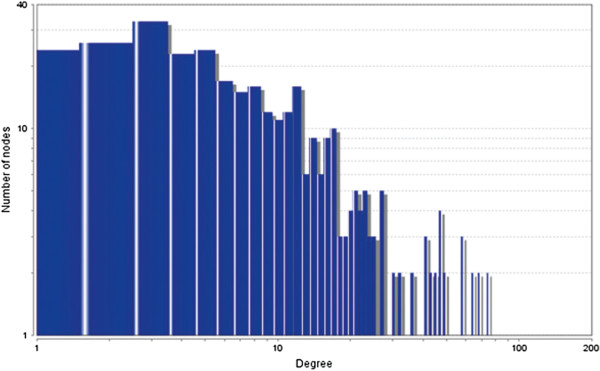
Degree distribution in bone cancer network.

**Figure 31:**
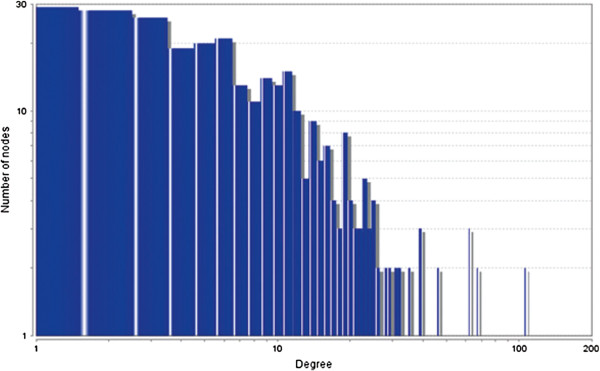
Degree distribution in breast normal network.

**Figure 32:**
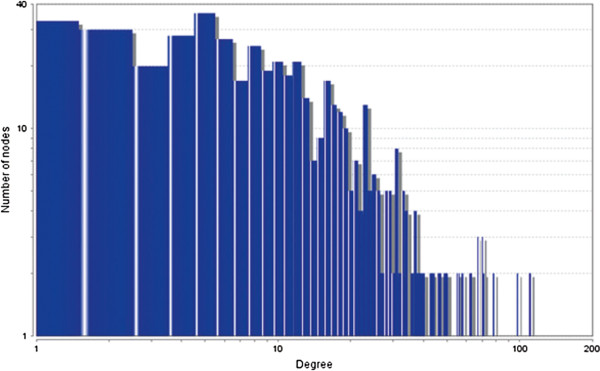
Degree distribution in breast cancer network.

**Figure 33:**
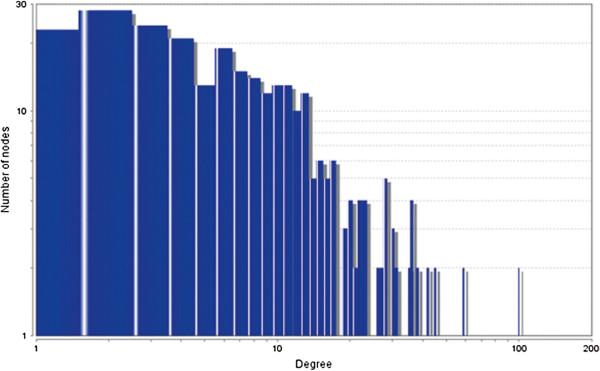
Degree distribution in colon normal network.

**Figure 34:**
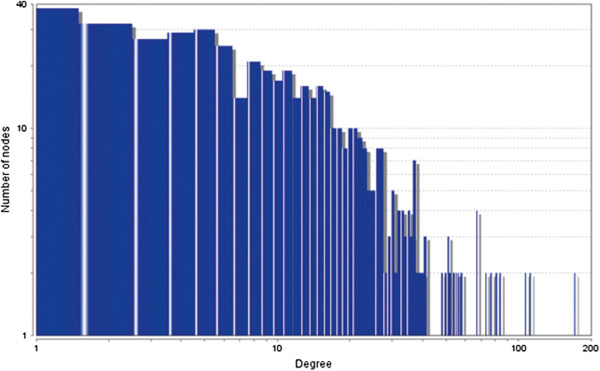
Degree distribution in colon cancer network.

**Figure 35:**
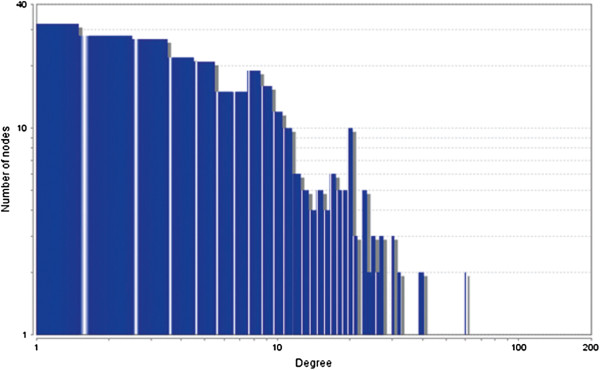
Degree distribution in kidney normal network.

**Figure 36:**
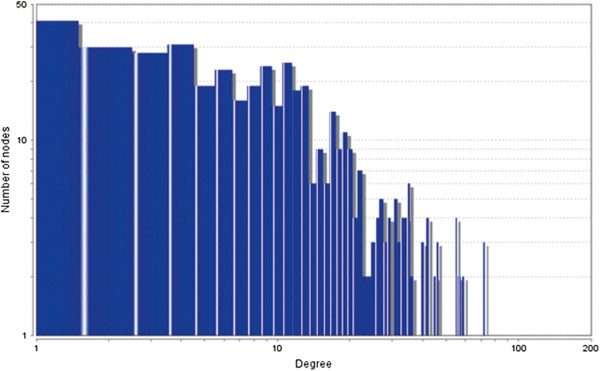
Degree distribution in kidney cancer network.

**Figure 37:**
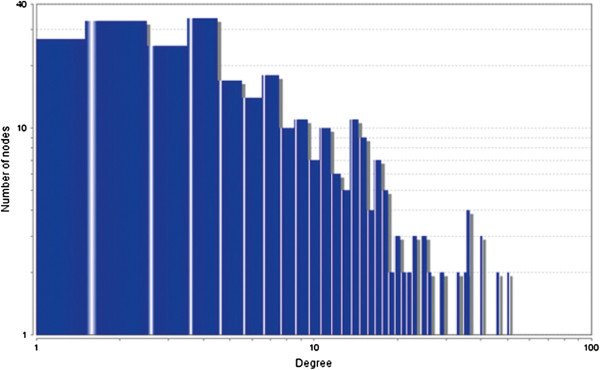
Degree distribution in liver normal network.

**Figure 38:**
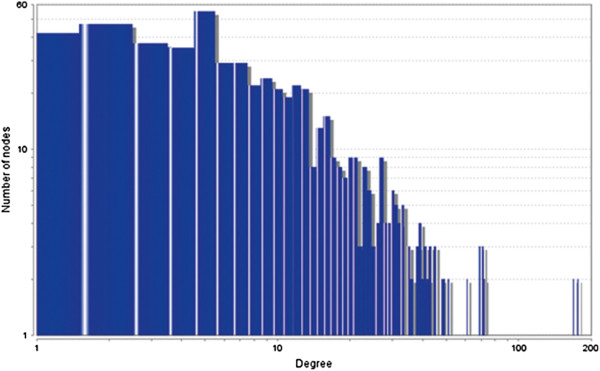
Degree distribution in liver cancer network.

The MCODE study shows that during cancer condition in each tissue, network clustering is increased. The ModuLand study denotes that the crucial nodes with module centrality are decreased in cancer (except breast cancer) representing the reduced level of module overlapping of cancer networks. The possible reason can be explained by degree distribution of the networks (Figures 29, 30, 31, 32, 33, 34, 35, 36, 37 and 38). Degree distribution of the networks can account for a possible explanation for counter behaving such clustering and overlapping. In all cancer PINs, few selective nodes with much higher degree are found contrary to the normal PINs. From this observation, a plausible argument can be proposed that some giant nodes are formed in cancer PINs covering a huge degree number and result in many randomly dispersed nodes. Such instance reduces the number of nodes with module centrality and subsequently overlapping modules with reduced number of nodes and edges are formed.

## Conclusion

The study gives us a clear picture of the differential modular nature between normal and cancer protein interaction networks. Normal and cancer protein interaction networks (PINs) show observable differences in case of both molecular complex and crucial node identification. The cancer PINs show higher predicted clustering but lower overlapping of network modules in contrast to the normal ones. The changes in predicted molecular complexes between normal and cancer PINs can be a handy tool to decipher the conversion of normal cells to cancer cells. The major molecular complexes (higher ranked) resulted from this study can be merged with experimental evidences to identify the core regulators responsible for cancer enigma. The identified crucial nodes can be recommended as potential drug targets against cancer and can be further assessed with experimental studies. This study can be further intensified through the inclusion of whole proteomic networks for normal and cancer cells derived from high throughput proteomic methods and their subsequent analysis by comprehensive computational tools. The networks considered here are unweighted and static which makes it less reliable to understand the real dynamic physical nature of living tissues. So it requires further expedition to comprehend the dynamics as well as to overcome the present limitations of network level understanding of biological processes. Moreover, the protein interaction study has to be merged with corresponding gene regulatory networks to draw more authentic conclusion regarding predicted modularity.

## Competing interests

The authors declare that they have no competing interests.

## Supplementary Material

Additional file 1 Differential protein interaction networks for normal and cancer tissues. Click here for file

Additional file 2 Expression and interaction data related to normal and cancer conditions of five tissues. Click here for file

Additional file 3 Tables for statistical significant test. Click here for file

Additional file 4 List of proteins of molecular complexes in normal and cancer protein interaction networks. Click here for file
