# Supplementary material for: Comparative analysis of differential network modularity in tissue specific normal and cancer protein interaction networks
Source: J Clin Bioinforma. 2013 Oct 6;3:19. doi: 10.1186/2043-9113-3-19 (PMC3852839; doi:10.1186/2043-9113-3-19)
Supplement: Additional file 3 — Tables for statistical significant test. [file 2043-9113-3-19-S3.pdf]

**Table 1- Molecular Cluster number difference between normal and cancer condition**

| Tissue | Normal | Cancer |
|---|---|---|
| Bone | 15 | 19 |
| Breast | 22 | 28 |
| Colon | 22 | 27 |
| Kidney | 21 | 30 |
| Liver | 19 | 28 |
| *p*-value | | 0.024658652 |

**Table 2- Interaction number difference of overlapping modules between normal and cancer condition**

| Tissue | Normal | Cancer |
|---|---|---|
| Breast | 5 | 3 |
| Colon | 5 | 5 |
| Kidney | 9 | 2 |
| Liver | 4 | 1 |
| *p*-value | | 0.07873529 |
